# Development and internal validation of an inflammation-platelet synergy score for anterior circulation cerebral infarction: an exploratory case-control study

**DOI:** 10.3389/fneur.2026.1805305

**Published:** 2026-07-16

**Authors:** Hanmei Cui, Ziwei Xu, Ting Cao, Yan Wang, Jingyan Zhang

**Affiliations:** 1 The Sixth Division of the Department of Neurology, The Second Affiliated Hospital of Qiqihar Medical College, Qiqihar, Heilongjiang, China; 2 The Fifth Division of the Department of Cardiovascular, The Second Affiliated Hospital of Qiqihar Medical College, Qiqihar, Heilongjiang, China; 3 College of Pathology, Qiqihar Medical University, Qiqihar, Heilongjiang, China

**Keywords:** anterior circulation cerebral infarction, carotid atherosclerosis, C-reactive protein, immunothrombosis, mean platelet volume, risk stratification

## Abstract

**Background:**

Inflammatory and thrombotic pathways interact in atherothrombotic stroke pathogenesis, yet combined assessment of these markers for distinguishing patients with anterior circulation cerebral infarction from high-risk controls remains underexplored. We aimed to develop and internally validate an inflammation-platelet synergy score in an exploratory case-control framework.

**Methods:**

This retrospective case-control study enrolled 203 patients with anterior circulation cerebral infarction and 98 high-risk controls with carotid atherosclerosis from January 2018 to June 2024. The synergy score incorporated six parameters: C-reactive protein (CRP), mean platelet volume (MPV), platelet distribution width (PDW), platelet count, intima-media thickness (IMT), and unstable plaque status. Discriminative performance was assessed using receiver operating characteristic analysis with bootstrap internal validation. Decision curve analysis was performed to evaluate potential clinical utility. Risk stratification was evaluated across low-risk (0–2 points), intermediate-risk (3–4 points), and high-risk (5–7 points) categories.

**Results:**

Cases exhibited significantly elevated CRP (median 6.2 vs. 3.5 mg/L, *p* < 0.001), MPV (10.64 vs. 9.64 fL, *p* < 0.001), and IMT (1.16 vs. 0.90 mm, *p* < 0.001) compared with controls. The synergy score demonstrated good discriminative ability (AUC = 0.824; 95% CI: 0.781–0.867; optimism-corrected AUC = 0.812), outperforming all individual markers (AUC range: 0.582–0.762). Decision curve analysis demonstrated positive net benefit across a range of clinically relevant threshold probabilities (20–60%). Cerebral infarction prevalence increased progressively from 36.0% in low-risk to 87.2% in high-risk groups (OR = 12.10; 95% CI: 5.40–27.15; *p* < 0.001). In exploratory secondary analyses among cases, higher scores were associated with greater stroke severity (Spearman rho = 0.312, *p* < 0.001) and unfavorable functional outcome at discharge (51.6% vs. 85.3% for low-risk vs. high-risk; P for trend <0.001).

**Conclusion:**

In this exploratory case-control study, the inflammation-platelet synergy score demonstrated good discriminative ability for anterior circulation cerebral infarction using routinely available parameters. These preliminary findings require prospective validation with pre-event biomarker measurement before any clinical application can be considered.

## Introduction

Stroke remains the second leading cause of mortality and third leading cause of disability-adjusted life years lost worldwide, with ischemic stroke accounting for approximately 62% of all incident strokes (1). The Global Burden of Disease Study 2019 documented 12.2 million incident strokes, 101 million prevalent strokes, and 6.55 million stroke-related deaths globally (2). Anterior circulation cerebral infarction, involving territories supplied by the internal carotid artery and its branches, represents the most common ischemic stroke subtype and frequently results in substantial neurological deficits including hemiparesis, aphasia, and cognitive impairment (3, 4).

The pathogenesis of ischemic stroke involves complex interactions among vascular, metabolic, and immunological mechanisms that contribute to atherosclerotic plaque formation, destabilization, and thromboembolism (5). Atherosclerosis is now recognized as a chronic inflammatory disease characterized by lipid accumulation, immune cell infiltration, and progressive fibrous cap formation within the arterial intima (6). Contemporary risk assessment has shifted from stenosis-centric approaches toward vulnerability-focused evaluation, recognizing that plaque composition and morphology; rather than luminal narrowing alone, are important determinants of clinical outcomes (7, 8). Vulnerable plaques characterized by large lipid-rich necrotic cores, thin fibrous caps, and intraplaque hemorrhage demonstrate heightened rupture propensity (9).

Systemic inflammation plays a key role throughout the atherosclerotic disease continuum. C-reactive protein (CRP), synthesized hepatically in response to interleukin-6, is the most extensively studied inflammatory biomarker in cardiovascular medicine (10, 11). The Emerging Risk Factors Collaboration analysis of 160,309 participants demonstrated continuous associations between CRP concentration and coronary heart disease, ischemic stroke, and vascular mortality (12, 13). The CANTOS trial provided proof-of-concept evidence that targeted anti-inflammatory therapy with canakinumab reduces recurrent cardiovascular events independent of lipid modification (14).

Beyond serving as a risk marker, CRP may participate in atherothrombus pathogenesis through complement activation, tissue factor induction, and enhanced platelet reactivity (15, 16). Activated platelets can amplify inflammation through release of cytokines including interleukin-1β, CD40 ligand, and platelet factor 4 (17, 18). This bidirectional relationship forms the conceptual basis for immunothrombosis—a paradigm in which innate immune activation and thrombus formation are mechanistically linked through shared molecular mediators and cellular interactions (19). In the context of atherosclerotic plaque rupture, immunothrombosis may drive both local thrombus propagation and systemic amplification of the prothrombotic state, suggesting that combined assessment of inflammatory and platelet markers may capture complementary pathogenic information.

Platelet morphological indices derived from routine complete blood count have emerged as accessible markers of platelet reactivity. Mean platelet volume (MPV) reflects average platelet size and correlates with functional activity, as larger platelets contain greater quantities of dense granules and pro-aggregatory surface receptors (20). Elevated MPV has been associated with increased risks of acute coronary syndromes, ischemic stroke, and cardiovascular mortality (21–23). Platelet distribution width (PDW) measures heterogeneity in platelet size and has been linked to adverse outcomes in acute ischemic stroke (24–26).

Carotid intima-media thickness (IMT), quantifiable through B-mode ultrasonography, serves as a validated surrogate marker of subclinical atherosclerosis (27). The ARIC, CHS, and Rotterdam cohort studies demonstrated that each 0.1-mm increment in common carotid IMT confers approximately 15–18% increased relative risk for incident stroke (28, 29). Characterization of plaque morphology provides additional prognostic information beyond IMT measurement alone (30).

While individual inflammatory and platelet biomarkers have been extensively investigated, their combined discriminative utility remains underexplored. Liu and colleagues recently demonstrated that high-sensitivity CRP modifies the prognostic value of platelet count in ischemic stroke (31). Building on this work, the present study aimed to develop and internally validate an inflammation-platelet synergy score for distinguishing patients with anterior circulation cerebral infarction from high-risk controls with carotid atherosclerosis. Given the retrospective case-control design, this study is exploratory in nature and intended to provide preliminary evidence for the combined assessment approach, with the understanding that prospective validation with pre-event biomarker measurement would be required before any clinical application.

## Materials and methods

### Study design and population

This retrospective case-control study was conducted at the Department of Neurology, Second Affiliated Hospital of Qiqihar Medical University, between January 2018 and June 2024. The study was designed as an exploratory biomarker discovery and internal validation investigation to develop a candidate composite score for distinguishing anterior circulation cerebral infarction cases from high-risk controls; because no intervention was tested and only routinely collected clinical data were analyzed retrospectively, the study did not meet criteria requiring clinical trial registration. The prespecified primary performance metric was the area under the receiver operating characteristic curve (AUC) for the composite score.

The case group comprised 203 patients with confirmed anterior circulation cerebral infarction. Consecutive patients meeting eligibility criteria were identified through electronic medical record review. Case inclusion criteria were:

- Age ≥18 years
- Acute ischemic stroke in anterior circulation territories (middle cerebral artery, anterior cerebral artery, or internal carotid artery distributions) confirmed by diffusion-weighted MRI or non-contrast CT within 72 h of symptom onset
- Presence of ipsilateral carotid atherosclerotic plaque on ultrasound examination
- Complete laboratory data obtained within 24 h of admission

The control group included 98 high-risk individuals with documented carotid atherosclerosis who did not experience cerebral infarction during the study period. Controls were identified from outpatient neurology and vascular medicine clinics during the same time frame and were frequency-matched to cases by 5-year age strata and sex to minimize confounding. Control subjects met criteria for carotid atherosclerosis (IMT ≥ 0.9 mm or visible plaque on ultrasonography) with at least one conventional vascular risk factor (hypertension, diabetes mellitus, dyslipidemia, or current smoking) but without clinical or neuroimaging evidence of prior or incident cerebral infarction. The selection of high-risk controls with carotid atherosclerosis, rather than healthy subjects, was a deliberate design choice intended to evaluate the score’s ability to discriminate among individuals with established vascular disease — a clinically relevant scenario where risk stratification is most needed.

Exclusion criteria for both groups were:

- Posterior circulation stroke (vertebrobasilar territory)
- Cardioembolic stroke etiology per TOAST classification (atrial fibrillation, mechanical valve, recent myocardial infarction, dilated cardiomyopathy)
- Active infection within 2 weeks preceding enrollment
- Known malignancy
- Primary hematological disorders affecting platelet count or function
- Chronic inflammatory or autoimmune diseases (rheumatoid arthritis, systemic lupus erythematosus, inflammatory bowel disease)
- Severe hepatic dysfunction (Child-Pugh class B or C) or renal dysfunction (eGFR <30 mL/min/1.73 m^2^)
- Current anticoagulant therapy

Sample size estimation was performed *a priori* using PASS 15.0 software. Based on pilot data suggesting an anticipated AUC of 0.80 for the composite score vs. 0.70 for individual markers, with *α* = 0.05 (two-sided) and 80% power, a minimum of 180 subjects per group was required for AUC comparison. The final sample of 301 subjects (203 cases, 98 controls) provided adequate statistical power for primary AUC analyses. However, the control group (*n* = 98) fell short of the estimated requirement of 180, reflecting practical constraints on recruitment of eligible high-risk controls during the study period.

This study was approved by the Institutional Ethics Committee of the Second Affiliated Hospital of Qiqihar Medical University (approval number: QMUE-2018-012). The study was conducted in accordance with the Declaration of Helsinki. Requirement for written informed consent was waived by the ethics committee due to the retrospective nature of the investigation and use of de-identified data.

### Clinical and laboratory assessment

Demographic characteristics (age, sex), medical history (hypertension, diabetes mellitus, dyslipidemia, smoking status), and medication use (antiplatelet agents, statins, antihypertensive medications) were extracted from electronic medical records by trained research personnel. Hypertension was defined as systolic blood pressure ≥140 mmHg, diastolic blood pressure ≥90 mmHg, or current antihypertensive medication use. Diabetes mellitus was defined as fasting plasma glucose ≥7.0 mmol/L, HbA1c ≥ 6.5%, or current hypoglycemic therapy. Dyslipidemia was defined as total cholesterol ≥5.2 mmol/L, LDL-cholesterol ≥3.4 mmol/L, triglycerides ≥1.7 mmol/L, or current lipid-lowering therapy. Current smoking was defined as regular cigarette use within the 12 months preceding enrollment.

Fasting venous blood samples were collected within 24 h of hospital admission (median 8.2 h from admission for cases) into EDTA-anticoagulated tubes for hematological analysis and serum separator tubes for biochemical testing. It should be noted that, given the retrospective case-control design, biomarkers in cases were measured after stroke onset and may therefore reflect acute-phase responses rather than solely pre-existing states. Complete blood count including platelet count, mean platelet volume (MPV), and platelet distribution width (PDW) was performed using the Sysmex XN-9000 automated hematology analyzer (Sysmex Corporation, Kobe, Japan) within 2 h of sample collection to minimize platelet swelling artifacts. The analyzer was calibrated daily according to manufacturer specifications; intra-assay coefficients of variation were <3% for platelet count and <2% for MPV.

C-reactive protein (CRP) was determined by particle-enhanced immunoturbidimetric assay using the Roche Cobas c702 analyzer (Roche Diagnostics, Basel, Switzerland) with a lower detection limit of 0.3 mg/L and measuring range of 0.3–350 mg/L. Inter-assay coefficient of variation was <5% across the clinical range.

### Carotid ultrasound examination

High-resolution B-mode carotid ultrasonography was performed by experienced vascular sonographers (each with >5 years of dedicated vascular ultrasound experience) using a Philips EPIQ 7 system (Philips Healthcare, Andover, MA) equipped with a 7.5–12 MHz linear array transducer. Sonographers were blinded to clinical and laboratory findings. All examinations were performed with standardized patient positioning (supine, neck extended, head rotated 45° contralaterally) and instrument settings (gain, depth, focal zone optimization).

Intima-media thickness (IMT) was measured at the far wall of the distal common carotid artery, 1–2 cm proximal to the carotid bifurcation, using automated edge-detection software. Three measurements were obtained on each side, and the mean of six measurements was recorded as the IMT value. Plaque was defined as a focal structure encroaching into the arterial lumen by at least 0.5 mm, or 50% of the surrounding IMT value, or demonstrating IMT > 1.5 mm, according to Mannheim Consensus criteria (32).

Plaque echogenicity was classified using the Gray-Weale classification system (33): Type I (predominantly echolucent with thin echogenic cap), Type II (substantially echolucent with <50% echogenic areas), Type III (predominantly echogenic with <50% echolucent areas), and Type IV (uniformly echogenic). Plaque surface morphology was classified as smooth, irregular, or ulcerated based on contour characteristics. Unstable plaque was defined as the presence of any of the following features: Type I or II echogenicity, irregular or ulcerated surface morphology, intraplaque hypoechoic zones suggesting hemorrhage or lipid accumulation, or maximum plaque thickness >4 mm.

All ultrasound examinations were reviewed by a second senior sonographer, with discrepancies resolved by consensus. Interobserver agreement was assessed in a random subset of 50 examinations: *κ* = 0.82 (95% CI: 0.71–0.93) for plaque stability classification; intraclass correlation coefficient (ICC) = 0.91 (95% CI: 0.85–0.95) for IMT measurement.

### Neurological assessment

Stroke severity at admission was assessed using the National Institutes of Health Stroke Scale (NIHSS), a validated 15-item instrument with scores ranging from 0 (no deficit) to 42 (most severe) (34). NIHSS assessments were performed by stroke-certified neurologists within 6 h of admission. Stroke severity was categorized as mild (NIHSS 0–4), moderate (5–15), or severe (≥16).

Functional outcome at discharge was evaluated using the modified Rankin Scale (mRS), a 7-level ordinal scale ranging from 0 (no symptoms) to 6 (death) (35). The mRS was assessed by neurologists blinded to biomarker results. Scores of 0–2 were defined as favorable outcome (functional independence), while scores of 3–6 were defined as unfavorable outcome (functional dependence or death).

### Inflammation-platelet synergy score development

The inflammation-platelet synergy scoring system was developed using a systematic, data-driven approach. First, receiver operating characteristic (ROC) curve analysis was performed for each candidate biomarker to determine optimal discrimination thresholds using the Youden index (J = sensitivity + specificity − 1). We acknowledge that dichotomization of continuous variables results in some loss of information and reduced statistical power; however, this approach was chosen to facilitate potential bedside application, as clinicians can more readily interpret binary thresholds than continuous scores in acute care settings. Second, candidate variables demonstrating significant independent associations with cerebral infarction status in multivariable logistic regression (*p* < 0.05) were retained for score construction. Third, point assignments (1 or 2 points) were determined based on the magnitude of adjusted regression coefficients, with variables showing *β*-coefficients in the upper tertile receiving 2 points and those in the lower two tertiles receiving 1 point. This approach follows the method described by Sullivan et al. for translating regression models into point-based scoring systems (36).

The final scoring system comprised six parameters: CRP > 3.0 mg/L (1 point), MPV > 10.2 fL (1 point), PDW > 15.5% (1 point), platelet count <150 × 10^9^/L (1 point), presence of unstable plaque (2 points), and IMT ≥ 1.0 mm (1 point). Total scores ranged from 0 to 7 points. Notably, low platelet count (<150 × 10^9^/L) did not achieve statistical significance in multivariable analysis (*p* = 0.234) but was retained in the scoring system based on pathophysiological considerations: relative thrombocytopenia in the context of acute atherothrombotic events may reflect platelet consumption at sites of active thrombus formation, a phenomenon well-documented in the immunothrombosis literature (19, 37). A sensitivity analysis comparing score performance with and without this component is presented in the Results. Risk stratification categories were defined based on observed cerebral infarction rates and clinical interpretability: low risk (0–2 points), intermediate risk (3–4 points), and high risk (5–7 points). Component thresholds and individual discriminative performance are presented in Table 1.

**Table 1:** Inflammation-platelet synergy scoring system.

Parameter	Threshold	Points	AUC (95% CI)
C-reactive protein	>3.0 mg/L	1	0.694 (0.633–0.755)
Mean platelet volume	>10.2 fL	1	0.718 (0.659–0.777)
Platelet distribution width	>15.5%	1	0.651 (0.587–0.715)
Platelet count	<150 × 10^9^/L	1	0.582 (0.515–0.649)
Unstable plaque	Present	2	0.693 (0.631–0.755)
Intima-media thickness	≥1.0 mm	1	0.762 (0.708–0.816)

Risk stratification: Low risk (0–2 points); Intermediate risk (3–4 points); High risk (5–7 points). Thresholds were determined by receiver operating characteristic curve analysis using the Youden index. Point assignments were based on regression coefficient magnitudes, following the approach of Sullivan et al. (43), with unstable plaque receiving 2 points due to its β-coefficient in the upper tertile. The platelet count component did not achieve statistical significance in multivariable analysis (*p* = 0.234). AUC, area under the receiver operating characteristic curve; CI, confidence interval.

### Statistical analysis

All statistical analyses were performed using SPSS version 26.0 (IBM Corporation, Armonk, NY) and R version 4.2.0 (R Foundation for Statistical Computing, Vienna, Austria). The significance threshold was set at two-sided *p* < 0.05 for all analyses.

Continuous variables were assessed for normality using the Shapiro–Wilk test, selected as appropriate for the per-group sample sizes (*n* = 98–203). Variables with Shapiro–Wilk *p* > 0.05 were considered normally distributed and presented as mean ± standard deviation (SD); variables with *p* ≤ 0.05 were considered non-normally distributed and presented as median with interquartile range (IQR: 25th–75th percentile). Categorical variables were presented as frequency and percentage.

Between-group comparisons of continuous variables were performed using independent-samples t-tests for normally distributed data and the Mann–Whitney U test for non-normally distributed data. For t-tests, homogeneity of variances was assessed using Levene’s test; Welch’s correction was applied when Levene’s test indicated heterogeneous variances (*p* < 0.05). Categorical variables were compared using Pearson’s chi-square test of independence; Fisher’s exact test was substituted when expected cell frequencies were <5.

Bivariate correlations among continuous variables were assessed using Spearman’s rank correlation coefficients (*ρ*) given the non-normal distribution of several variables and the ordinal nature of clinical outcome scales. Correlation strength was interpreted as negligible (|ρ| < 0.10), weak (0.10–0.29), moderate (0.30–0.49), or strong (≥0.50).

Multivariable binary logistic regression was performed to identify independent predictors of cerebral infarction status. Predictor variables were selected *a priori* based on established pathophysiological relevance and prior literature: age, sex, hypertension, diabetes mellitus, dyslipidemia, current smoking, statin use, CRP (dichotomized), MPV (dichotomized), PDW (dichotomized), platelet count (dichotomized), IMT (dichotomized), and unstable plaque status. All pre-specified predictors were entered simultaneously (forced entry method) rather than using stepwise selection to avoid inflated Type I error and overfitting. We acknowledge that body mass index (BMI) and additional hematological markers (e.g., neutrophil-to-lymphocyte ratio, fibrinogen) were not included; BMI was not systematically recorded in all electronic medical records during the study period, and additional hematological markers were beyond the predefined scope of this investigation.

Model assumptions were verified as follows: linearity in the logit for continuous predictors was assessed using the Box-Tidwell test; multicollinearity was evaluated using variance inflation factors (VIF), with VIF > 5 indicating problematic collinearity; influential observations were identified using Cook’s distance (threshold > 4/n). No violations of model assumptions were detected (all VIF < 2.5; all Cook’s distances < 0.05; Box-Tidwell *p* > 0.05 for all continuous predictors). Model goodness-of-fit was assessed using the Hosmer-Lemeshow test, with *p* > 0.05 indicating adequate fit. Model calibration was additionally evaluated using calibration plots of observed vs. predicted probabilities. Overall model performance was quantified using Nagelkerke pseudo-R^2^.

Results are reported as odds ratios (OR) with 95% confidence intervals (CI) calculated using the Wald method. Effect sizes for group comparisons were quantified using Cohen’s d for continuous variables (small: 0.2; medium: 0.5; large: 0.8), phi coefficient (*φ*) for 2 × 2 categorical comparisons, and rank-biserial correlation (r) for non-parametric comparisons.

ROC curve analysis was performed to evaluate the discriminative ability of individual biomarkers and the composite synergy score. The area under the ROC curve (AUC) was calculated with 95% CIs derived using the DeLong method. AUC values were interpreted as follows: 0.50–0.59 = no discrimination, 0.60–0.69 = poor, 0.70–0.79 = acceptable, 0.80–0.89 = excellent, ≥0.90 = outstanding. We note that AUC values derived from case-control studies may overestimate discriminative performance relative to prospective cohort settings due to potential spectrum bias and the fixed case-control ratio. Pairwise comparisons of AUCs between the synergy score and individual markers were performed using the DeLong test with Bonferroni correction for multiple comparisons (adjusted *α* = 0.05/6 = 0.008 for six pairwise comparisons). Optimal cutoff values were determined by maximizing the Youden index. Sensitivity, specificity, positive predictive value (PPV), negative predictive value (NPV), and diagnostic accuracy were calculated at the optimal threshold.

Internal validation was performed using bootstrap resampling with 1,000 iterations to estimate optimism-corrected performance metrics. The bootstrap procedure involved: (1) drawing a random sample with replacement from the original dataset, (2) developing the model in the bootstrap sample, (3) evaluating performance in both the bootstrap sample and original dataset, and (4) calculating the optimism as the difference between these estimates. The optimism-corrected AUC was calculated by subtracting the mean optimism from the apparent AUC. Calibration slope was estimated from the bootstrap procedure, with values near 1.0 indicating minimal overfitting. The events-per-variable (EPV) ratio was calculated as the number of events (cases) divided by the number of predictor variables to assess the adequacy of model complexity relative to sample size.

Decision curve analysis (DCA) was performed to evaluate the potential clinical utility of the synergy score by quantifying net benefit across a range of threshold probabilities (38). Net benefit was calculated as: net benefit = (true positives/n) − (false positives/n) × (threshold probability / [1 − threshold probability]). The DCA compared the synergy score against strategies of treating all patients and treating no patients. This analysis addresses whether use of the synergy score could offer a favorable balance between correctly identifying cases and avoiding unnecessary interventions, though clinical utility would need to be confirmed in prospective settings.

A sensitivity analysis was conducted to assess the impact of excluding the platelet count component (which did not achieve statistical significance in multivariable analysis) on the overall discriminative performance of the synergy score.

Trends in cerebral infarction incidence and unfavorable outcome rates across ordered risk categories were assessed using the Cochran-Armitage test for trend. Odds ratios comparing intermediate-risk and high-risk groups to the low-risk reference group were calculated with 95% CIs.

## Results

### Baseline characteristics

A total of 301 subjects were enrolled, comprising 203 patients with anterior circulation cerebral infarction and 98 high-risk controls with carotid atherosclerosis (Table 2). The case group was slightly older than the control group, with a mean age of 66.9 ± 10.3 vs. 64.1 ± 10.9 years (t-test: t = 2.13; df = 299; *p* = 0.034; Cohen’s d = 0.26, 95% CI: 0.02–0.51). Sex distribution did not differ significantly between groups, with males comprising 52.7% of cases and 61.2% of controls (χ^2^ test: χ^2^ = 1.98; df = 1; *p* = 0.160; *φ* = 0.08, 95% CI: −0.03–0.19).

**Table 2:** Baseline characteristics.

Variable	Case (*n* = 203)	Control (*n* = 98)	Test statistic	*p*	Effect size (95% CI)
Age, years	66.9 ± 10.3	64.1 ± 10.9	t = 2.13; df = 299	0.034	d = 0.26 (0.02–0.51)
Male, n (%)	107 (52.7)	60 (61.2)	χ^2^ = 1.98; df = 1	0.160	*φ* = 0.08 (−0.03–0.19)
Hypertension, n (%)	148 (72.9)	70 (71.4)	χ^2^ = 0.07; df = 1	0.790	φ = 0.02 (−0.10–0.13)
Diabetes mellitus, n (%)	87 (42.9)	25 (25.5)	χ^2^ = 8.69; df = 1	0.003	φ = 0.17 (0.06–0.28)
Dyslipidemia, n (%)	98 (48.3)	43 (43.9)	χ^2^ = 0.53; df = 1	0.470	φ = 0.04 (−0.07–0.15)
Current smoking, n (%)	79 (38.9)	36 (36.7)	χ^2^ = 0.14; df = 1	0.706	φ = 0.02 (−0.09–0.13)
Statin use, n (%)	63 (31.0)	34 (34.7)	χ^2^ = 0.40; df = 1	0.527	φ = 0.04 (−0.08–0.15)
CRP, mg/L*	6.2 (3.1–11.8)	3.5 (2.1–5.4)	U = 5,765	<0.001	r = 0.38 (0.27–0.48)
MPV, fL	10.64 ± 1.30	9.64 ± 1.08	t = 6.86; df = 299	<0.001	d = 0.84 (0.58–1.09)
PDW, %	15.13 ± 2.76	13.71 ± 2.08	t = 4.98; df = 237.4†	<0.001	d = 0.58 (0.33–0.83)
Platelet count, × 10^9^/L	217.2 ± 62.6	233.5 ± 45.3	t = −2.56; df = 254.8†	0.010	d = 0.30 (0.05–0.54)
IMT, mm	1.16 ± 0.28	0.90 ± 0.21	t = 8.71; df = 243.6†	<0.001	d = 1.05 (0.79–1.31)
Unstable plaque, n (%)	132 (65.0)	26 (26.5)	χ^2^ = 39.04; df = 1	<0.001	φ = 0.36 (0.25–0.46)

Data presented as mean ± SD for normally distributed continuous variables, median (IQR) for non-normally distributed continuous variables, or n (%) for categorical variables. Median (interquartile range) due to non-normal distribution (Shapiro–Wilk *p* < 0.001). †Welch’s correction applied due to unequal variances (Levene’s test *p* < 0.05). Effect sizes: d, Cohen’s d; φ, phi coefficient; r, rank-biserial correlation. CI, confidence interval; CRP, C-reactive protein; IMT, intima-media thickness; MPV, mean platelet volume; PDW, platelet distribution width. * = non-normal variable, median (IQR), Mann-Whitney U test.

Regarding cardiovascular risk factors, diabetes mellitus was significantly more prevalent among cases than controls (42.9% vs. 25.5%; χ^2^ test: χ^2^ = 8.69; df = 1; *p* = 0.003; *φ* = 0.17, 95% CI: 0.06–0.28), whereas hypertension (72.9% vs. 71.4%; χ^2^ test: χ^2^ = 0.07; df = 1; *p* = 0.790; φ = 0.02, 95% CI: −0.10–0.13) and dyslipidemia (48.3% vs. 43.9%; χ^2^ test: χ^2^ = 0.53; df = 1; *p* = 0.470; *φ* = 0.04, 95% CI: −0.07–0.15) were comparably distributed between groups. Current smoking prevalence was similar between cases and controls (38.9% vs. 36.7%; χ^2^ test: χ^2^ = 0.14; df = 1; *p* = 0.706; φ = 0.02, 95% CI: −0.09–0.13). Statin use prior to enrollment was also comparable (31.0% vs. 34.7%; χ^2^ test: χ^2^ = 0.40; df = 1; *p* = 0.527; φ = 0.04, 95% CI: −0.08–0.15).

### Inflammatory and platelet parameters

**Figure 1:**
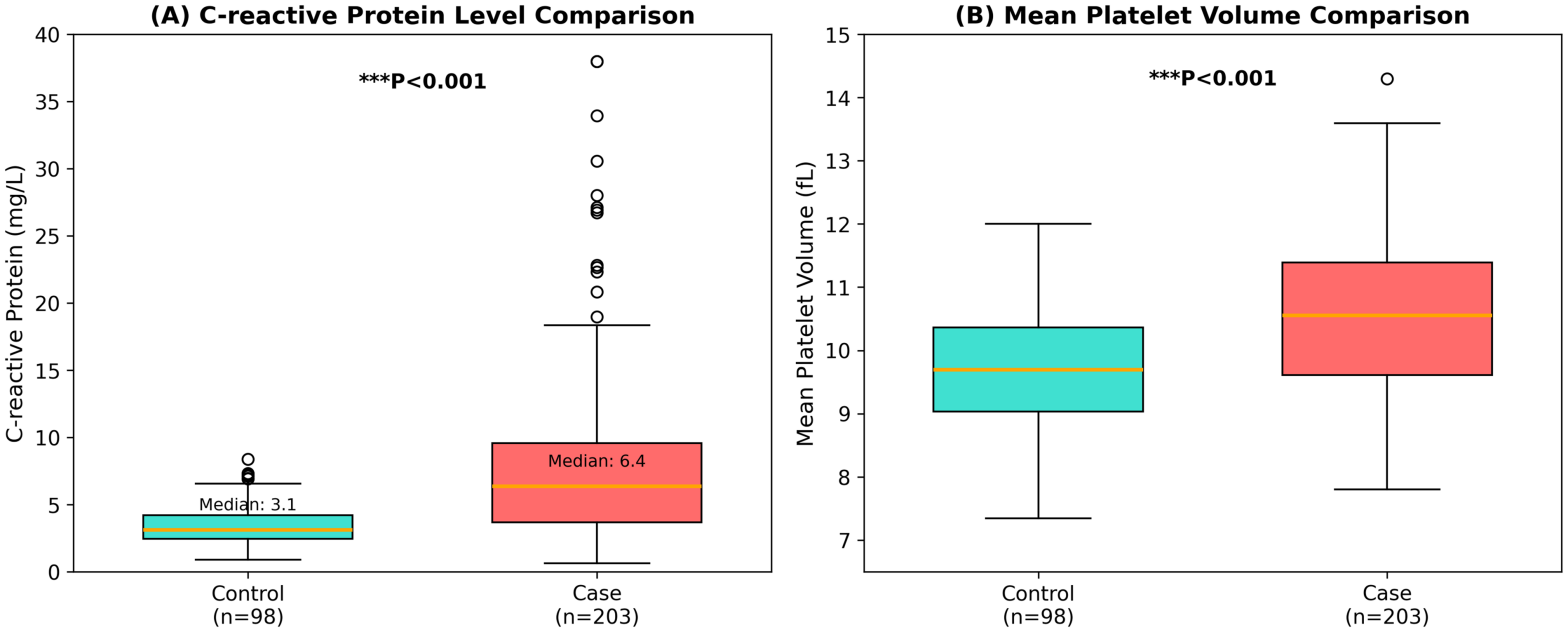
Comparison of C-reactive protein and mean platelet volume between groups. **(A)** C-reactive protein (CRP) levels in cases (*n* = 203) and controls (*n* = 98). **(B)** Mean platelet volume (MPV) in cases and controls. Box plots display median (horizontal line), interquartile range (box), range (whiskers), and outliers (points beyond 1.5 × IQR). CRP was compared using the Mann–Whitney U test due to non-normal distribution; MPV was compared using an independent-samples t-test. ***p < 0.001.

Cases exhibited significantly elevated inflammatory and platelet indices compared with controls (Table 2; Figure 1). C-reactive protein demonstrated markedly higher values in cases, with a median of 6.2 mg/L (IQR: 3.1–11.8) vs. 3.5 mg/L (IQR: 2.1–5.4) in controls (M–W test: U = 5,765; n = [203, 98]; *p* < 0.001; rank-biserial r = 0.38, 95% CI: 0.27–0.48). Mean platelet volume was elevated in cases at 10.64 ± 1.30 fL compared with 9.64 ± 1.08 fL in controls (t-test: t = 6.86; df = 299; *p* < 0.001; Cohen’s d = 0.84, 95% CI: 0.58–1.09), representing a large effect. Similarly, platelet distribution width was higher in cases (15.13 ± 2.76% vs. 13.71 ± 2.08%; t-test: t = 4.98; df = 237.4 [Welch-corrected]; *p* < 0.001; Cohen’s d = 0.58, 95% CI: 0.33–0.83).

In contrast, platelet count was modestly lower in the case group (217.2 ± 62.6 vs. 233.5 ± 45.3 × 10^9^/L; t-test: t = −2.56; df = 254.8 [Welch-corrected]; *p* = 0.010; Cohen’s d = 0.30, 95% CI: 0.05–0.54). Carotid intima-media thickness demonstrated the largest between-group difference, with cases exhibiting values of 1.16 ± 0.28 mm vs. 0.90 ± 0.21 mm in controls (t-test: t = 8.71; df = 243.6 [Welch-corrected]; *p* < 0.001; Cohen’s d = 1.05, 95% CI: 0.79–1.31). Unstable plaque was present in 65.0% of cases compared with 26.5% of controls (χ^2^ test: χ^2^ = 39.04; df = 1; *p* < 0.001; *φ* = 0.36, 95% CI: 0.25–0.46). Figure 1 displays the distributions of CRP (Panel A) and MPV (Panel B) by group.

### Correlation analysis

**Figure 2:**
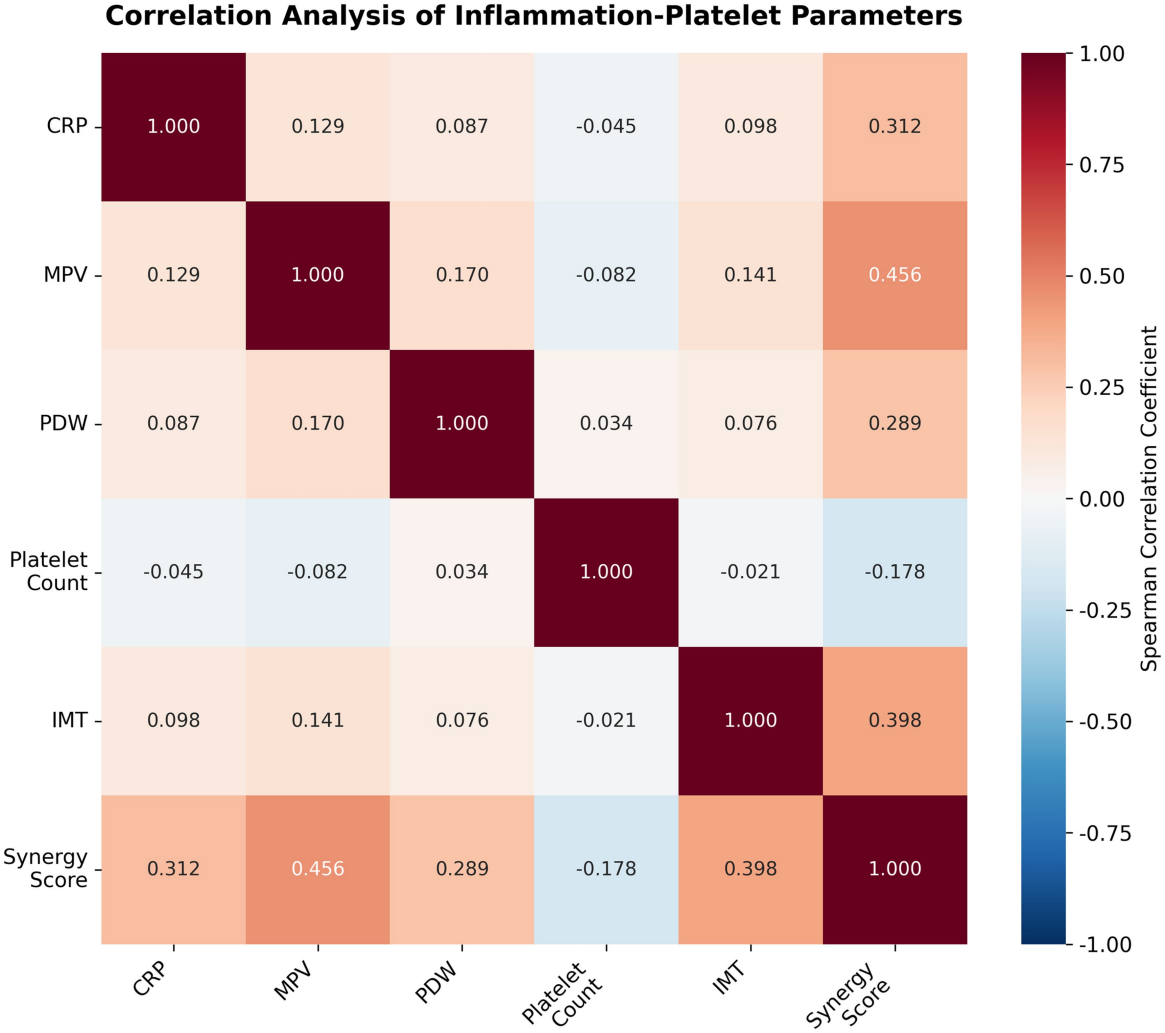
Spearman correlation matrix of inflammation-platelet parameters. Heatmap displaying Spearman correlation coefficients (*ρ*) among study variables including C-reactive protein (CRP), mean platelet volume (MPV), platelet distribution width (PDW), platelet count (PLT), intima-media thickness (IMT), unstable plaque status, synergy score, National Institutes of Health Stroke Scale (NIHSS), and modified Rankin Scale (mRS). Color intensity represents correlation magnitude: red indicates positive correlation; blue indicates negative correlation. Correlation coefficients are displayed within cells. *n* = 301 for biomarker correlations; *n* = 203 for correlations involving NIHSS and mRS (cases only).

Spearman correlation analysis revealed significant but modest intercorrelations among inflammation-platelet parameters (Figure 2). CRP demonstrated a weak positive correlation with MPV (*ρ* = 0.129; *n* = 301; *p* = 0.025). Stronger correlations were observed between MPV and PDW (*ρ* = 0.170; *n* = 301; *p* = 0.003) and between MPV and IMT (ρ = 0.141; n = 301; *p* = 0.014). The composite synergy score exhibited the strongest correlations with its component variables, particularly MPV (ρ = 0.456; *p* < 0.001) and IMT (ρ = 0.398; *p* < 0.001).

Regarding clinical outcomes, the synergy score demonstrated moderate positive correlations with both stroke severity and functional outcome among cases in exploratory unadjusted analyses. The correlation between synergy score and admission NIHSS was statistically significant (ρ = 0.312; *n* = 203; *p* < 0.001), as was the correlation with discharge mRS (ρ = 0.287; *n* = 203; *p* < 0.001). These correlations indicate that higher synergy scores are associated with greater neurological impairment at presentation and worse functional status at discharge, though the cross-sectional nature of these analyses limits causal interpretation.

### Multivariable logistic regression

**Figure 3:**
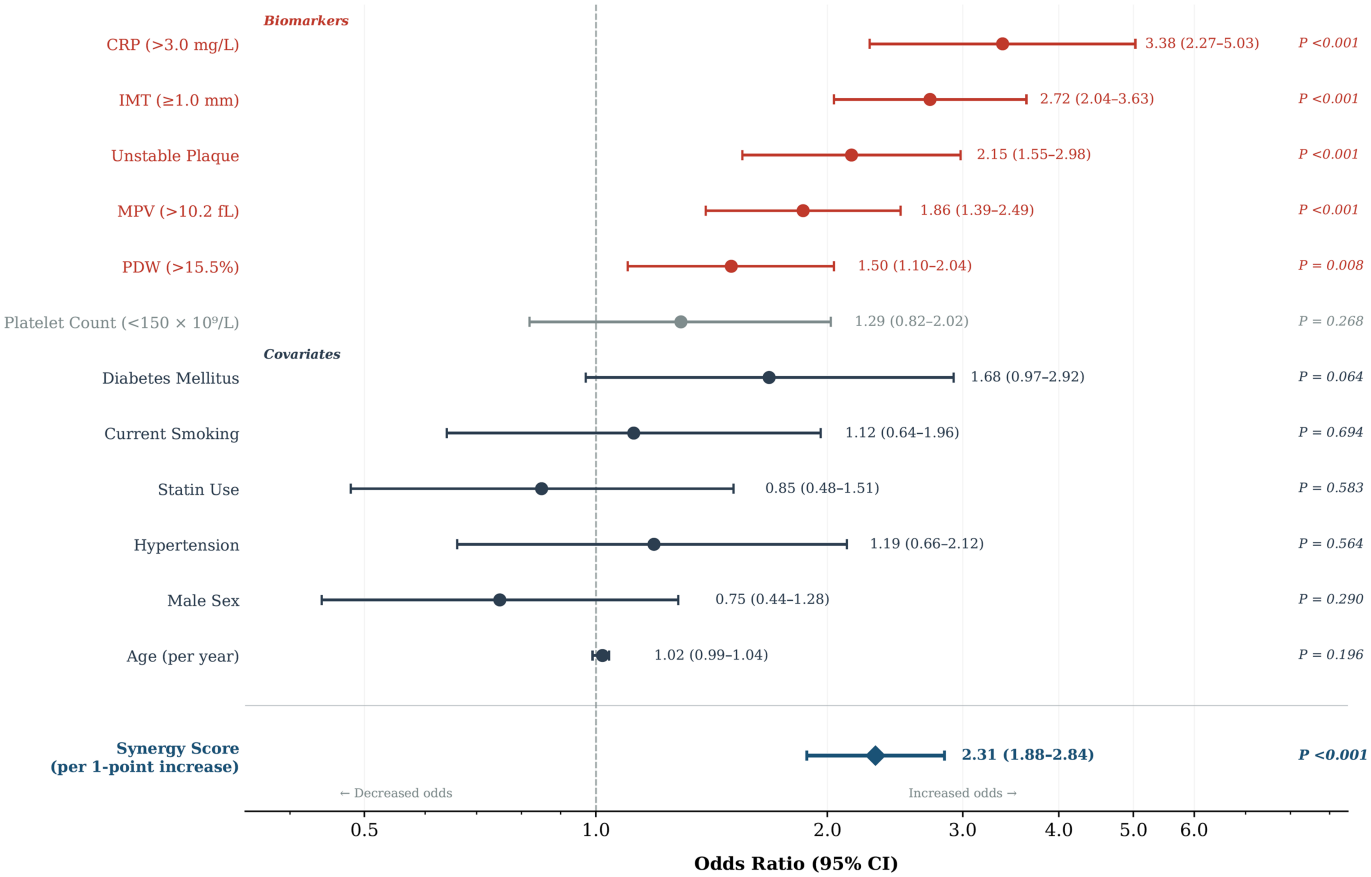
Forest plot of multivariable logistic regression analysis. Odds ratios (ORs) with 95% confidence intervals (CIs) for independent predictors of cerebral infarction. The model was adjusted for age, sex, hypertension, diabetes mellitus, dyslipidemia, current smoking, and statin use. Biomarkers were dichotomized at thresholds determined by receiver operating characteristic analysis (Table 1). The vertical dashed line indicates OR = 1.0 (null effect). ORs > 1.0 indicate increased odds of cerebral infarction. CRP, C-reactive protein; IMT, intima-media thickness; MPV, mean platelet volume; PDW, platelet distribution width. See Table 3 for complete regression statistics.

Multivariable logistic regression analysis, adjusted for age, sex, hypertension, diabetes mellitus, dyslipidemia, current smoking, and statin use, identified several independent predictors of cerebral infarction status (Table 3; Figure 3). The overall model demonstrated excellent fit (model χ^2^ = 144.3; df = 13; *p* < 0.001; Hosmer-Lemeshow χ^2^ = 8.56; df = 8; *p* = 0.381; Nagelkerke R^2^ = 0.53; classification accuracy = 79.7%). No multicollinearity was detected among predictors (all VIF < 2.5). The events-per-variable ratio was 203/13 = 15.6, exceeding the commonly recommended minimum of 10, though some methodologists recommend higher thresholds.

**Table 3:** Multivariable logistic regression analysis for cerebral infarction.

Variable	B	SE	95% CI (B)	OR	95% CI (OR)	*p*
Age (per year)	0.02	0.01	−0.01–0.04	1.02	0.99–1.04	0.196
Male sex	−0.29	0.28	−0.83–0.25	0.75	0.44–1.28	0.290
Hypertension	0.17	0.30	−0.41–0.75	1.19	0.66–2.12	0.564
Diabetes mellitus	0.52	0.28	−0.03–1.07	1.68	0.97–2.92	0.064
Dyslipidemia	0.11	0.27	−0.41–0.63	1.12	0.66–1.88	0.673
Current smoking	0.11	0.28	−0.44–0.67	1.12	0.64–1.96	0.694
Statin use	−0.16	0.29	−0.73–0.41	0.85	0.48–1.51	0.583
CRP > 3.0 mg/L	1.22	0.20	0.82–1.61	3.38	2.27–5.03	<0.001
MPV > 10.2 fL	0.62	0.15	0.33–0.91	1.86	1.39–2.49	<0.001
PDW > 15.5%	0.41	0.16	0.10–0.71	1.50	1.10–2.04	0.008
Platelet count <150 × 10^9^/L	0.25	0.23	−0.20–0.71	1.29	0.82–2.02	0.268
IMT ≥ 1.0 mm	1.00	0.15	0.71–1.29	2.72	2.04–3.63	<0.001
Unstable plaque	0.77	0.17	0.44–1.09	2.15	1.55–2.98	<0.001
Synergy score (per point)‡	0.84	0.11	0.63–1.05	2.31	1.88–2.84	<0.001

Model statistics (Individual Biomarkers Model): Overall model: χ^2^ = 144.3; df = 13; *p* < 0.001. Goodness-of-fit: Hosmer-Lemeshow χ^2^ = 8.56; df = 8; *p* = 0.381. Nagelkerke R^2^ = 0.53. Classification accuracy = 79.7%. Events-per-variable ratio = 203/13 = 15.6. ‡Synergy score analyzed in a separate model adjusted for age, sex, hypertension, diabetes mellitus, dyslipidemia, current smoking, and statin use (model χ^2^ = 100.2; df = 8; *p* < 0.001; Nagelkerke R^2^ = 0.42). Binary logistic regression with forced entry of all predictors. Outcome variable: cerebral infarction status (case = 1, control = 0). B, unstandardized regression coefficient (log-odds); SE, standard error; OR, odds ratio; CI, confidence interval.

Among dichotomized biomarkers, elevated CRP (>3.0 mg/L) exhibited the strongest independent association with cerebral infarction (OR = 3.38; 95% CI: 2.27–5.03; *p* < 0.001). Increased IMT (≥1.0 mm) conferred substantially elevated odds (OR = 2.72; 95% CI: 2.04–3.63; *p* < 0.001), followed by unstable plaque presence (OR = 2.15; 95% CI: 1.55–2.98; *p* < 0.001). Elevated MPV (>10.2 fL) was independently associated with cerebral infarction (OR = 1.86; 95% CI: 1.39–2.49; *p* < 0.001), as was elevated PDW (>15.5%; OR = 1.50; 95% CI: 1.10–2.04; *p* = 0.008). Low platelet count (<150 × 10^9^/L) did not reach statistical significance as an independent predictor (OR = 1.29; 95% CI: 0.82–2.02; *p* = 0.268), likely reflecting the low prevalence of thrombocytopenia in this cohort (8.6% of all subjects). Neither current smoking (OR = 1.12; 95% CI: 0.64–1.96; *p* = 0.694) nor statin use (OR = 0.85; 95% CI: 0.48–1.51; *p* = 0.583) achieved statistical significance, consistent with frequency matching on smoking status and comparable baseline medication use.

In a separate model evaluating the composite synergy score as a continuous predictor (adjusted for age, sex, hypertension, diabetes mellitus, dyslipidemia, current smoking, and statin use), each one-point increment was associated with a 2.31-fold increase in the odds of cerebral infarction (OR = 2.31; 95% CI: 1.88–2.84; *p* < 0.001; Table 3).

### Discriminative performance

**Figure 4:**
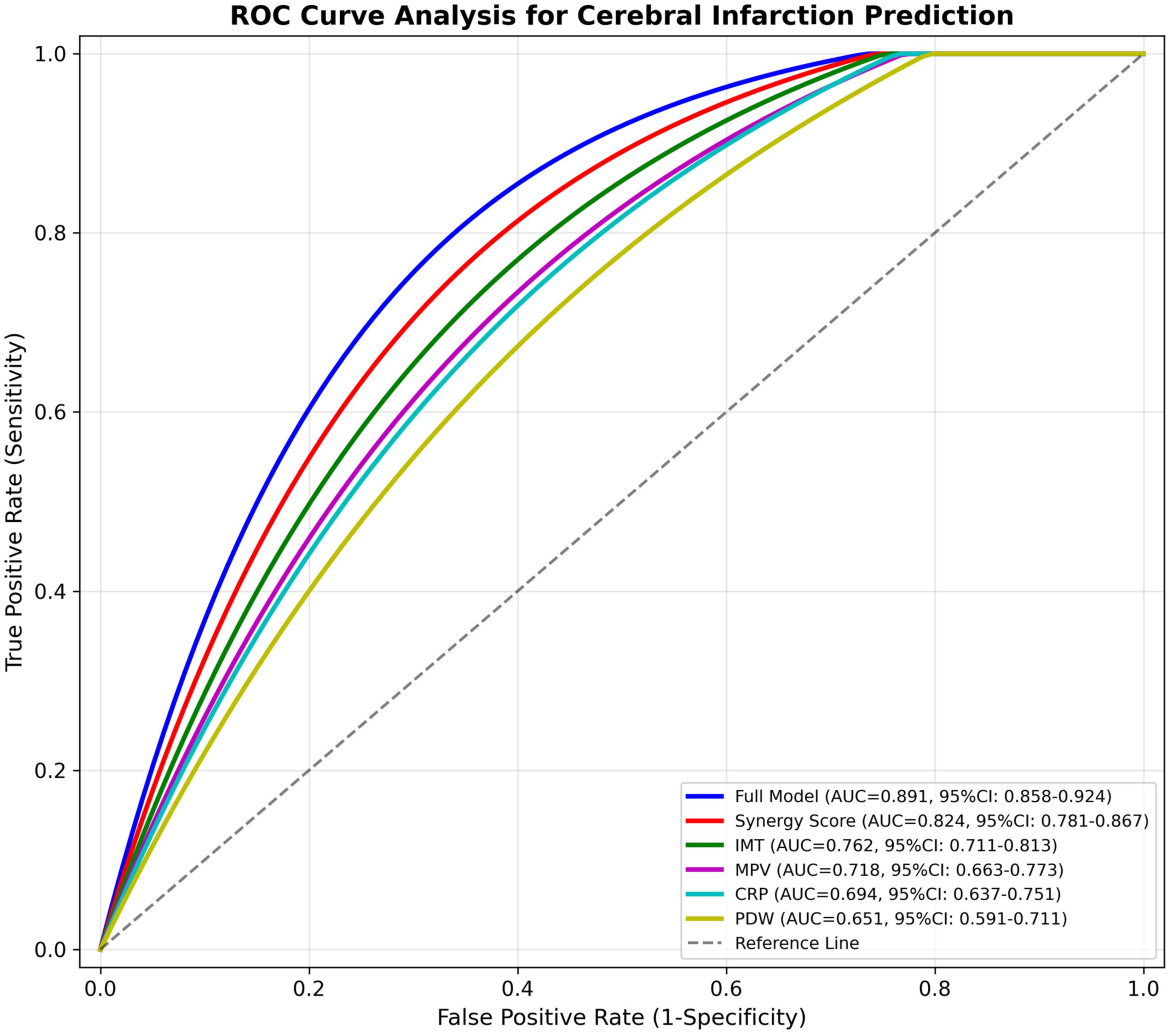
Receiver operating characteristic curves comparing synergy score and individual markers. Receiver operating characteristic (ROC) curves for the inflammation-platelet synergy score (solid black line) and individual biomarkers: intima-media thickness (IMT), mean platelet volume (MPV), C-reactive protein (CRP), unstable plaque, platelet distribution width (PDW), and platelet count. The diagonal gray line represents chance discrimination (AUC = 0.50). Area under the curve (AUC) values with 95% confidence intervals are shown in the legend. The synergy score (AUC = 0.824) demonstrated significantly greater discriminative ability than all individual markers (DeLong test with Bonferroni correction, all *p* < 0.008). *n* = 301. AUC estimates from case-control designs may overestimate prospective discriminative performance.

Receiver operating characteristic curve analysis demonstrated that the inflammation-platelet synergy score achieved good discriminative ability for identifying cerebral infarction (Figure 4). The synergy score yielded an AUC of 0.824 (95% CI: 0.781–0.867), which was superior to all individual biomarkers. The full multivariable model incorporating the synergy score along with demographic and clinical covariates achieved an AUC of 0.891 (95% CI: 0.858–0.924). We note that these AUC values should be interpreted with caution, as discriminative performance estimated in case-control studies may overestimate what would be observed in prospective cohort settings due to potential spectrum bias.

Among individual parameters (Table 1), IMT demonstrated the highest discriminative ability (AUC = 0.762; 95% CI: 0.708–0.816), followed by MPV (AUC = 0.718; 95% CI: 0.659–0.777), CRP (AUC = 0.694; 95% CI: 0.633–0.755), unstable plaque (AUC = 0.693; 95% CI: 0.631–0.755), PDW (AUC = 0.651; 95% CI: 0.587–0.715), and platelet count (AUC = 0.582; 95% CI: 0.515–0.649). DeLong test comparisons confirmed that the synergy score AUC was significantly greater than each individual marker (all Bonferroni-corrected *p* < 0.008). While comparing a composite score against its own components is inherently favorable to the composite, this analysis demonstrates that the combination captures discriminative information beyond any single marker.

**Figure 5:**
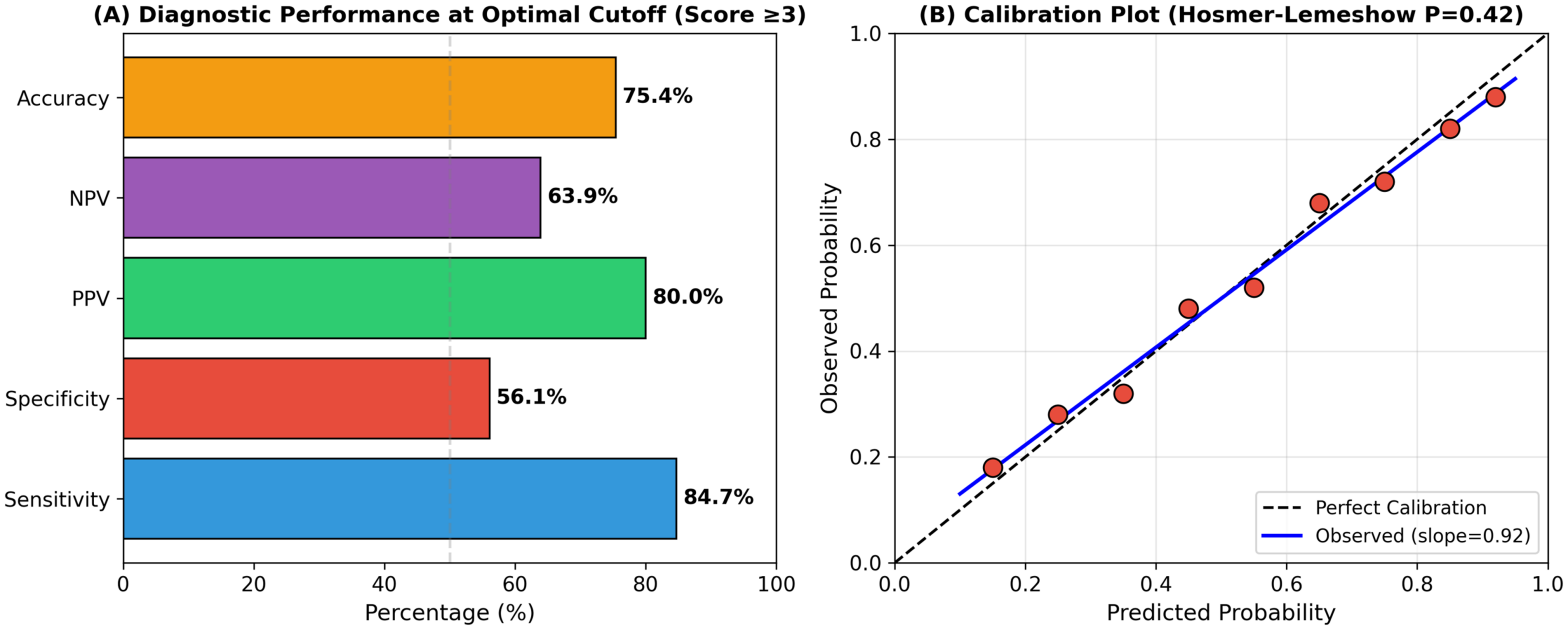
Diagnostic performance and model calibration. **(A)** Performance metrics for the synergy score at the optimal cutoff (≥3 points). Sensitivity = 84.7% (95% CI: 79.2–89.2%); specificity = 56.1% (95% CI: 45.7–66.1%); positive predictive value (PPV) = 80.0% (95% CI: 74.2–84.9%); negative predictive value (NPV) = 63.9% (95% CI: 53.2–73.6%); accuracy = 75.4% (95% CI: 70.2–80.2%). **(B)** Calibration plot showing observed vs. predicted probabilities of cerebral infarction. The diagonal dashed line represents perfect calibration. Points represent deciles of predicted probability. The solid line is the locally weighted scatterplot smoothing (LOWESS) fit. Calibration slope = 0.92; Hosmer-Lemeshow test: χ^2^ = 8.56, df = 8, *p* = 0.381. Bootstrap validation (1,000 iterations): optimism-corrected AUC = 0.812.

Bootstrap internal validation with 1,000 iterations yielded an optimism-corrected AUC of 0.812, indicating acceptable stability with modest overfitting (optimism = 0.012). At the optimal threshold of ≥3 points (determined by maximum Youden index), the synergy score demonstrated sensitivity of 84.7% (95% CI: 79.2–89.2%), specificity of 56.1% (95% CI: 45.7–66.1%), positive predictive value of 80.0% (95% CI: 74.2–84.9%), negative predictive value of 63.9% (95% CI: 53.2–73.6%), and overall diagnostic accuracy of 75.4% (95% CI: 70.2–80.2%; Figure 5A). The moderate specificity (56.1%) indicates that the score at this threshold would produce a substantial proportion of false positives, which may limit its utility as a standalone diagnostic tool; however, a high-sensitivity, moderate-specificity profile may be appropriate for a screening-oriented application where the priority is to avoid missing high-risk individuals, with subsequent confirmatory workup for those who screen positive. The calibration plot demonstrated good agreement between predicted probabilities and observed outcomes, with a calibration slope of 0.92 (Figure 5B).

### Decision curve analysis

**Figure 6:**
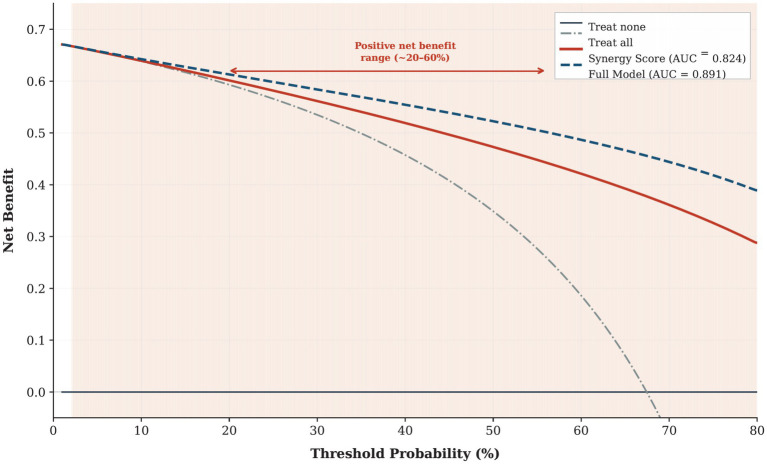
Decision curve analysis. Decision curve analysis comparing the net benefit of the inflammation-platelet synergy score (solid line) and the full multivariable model (dashed line) against strategies of treating all patients (gray line) and treating no patients (horizontal line at zero). The synergy score demonstrated positive net benefit across threshold probabilities of approximately 20 to 60%, suggesting potential clinical utility within this range. These estimates are derived from a case-control design and require prospective validation.

Decision curve analysis demonstrated that the synergy score provided positive net benefit compared with the strategies of treating all patients and treating no patients across a range of clinically relevant threshold probabilities from approximately 20 to 60% (Figure 6). The full multivariable model incorporating the synergy score with clinical covariates showed a slightly higher net benefit than the synergy score alone across this range. These findings suggest that, within this study population, the synergy score may offer a favorable balance between identifying true cases and avoiding unnecessary interventions, though the clinical utility estimates derived from this case-control study require prospective confirmation.

### Sensitivity analysis: score performance without platelet count

A sensitivity analysis excluding the platelet count component (resulting in a modified 5-parameter score, range 0–6) yielded an AUC of 0.819 (95% CI: 0.774–0.864), which was not significantly different from the full 6-parameter score (AUC = 0.824; DeLong *p* = 0.412). This finding confirms that the platelet count component contributes minimally to the overall discriminative performance. The component was nonetheless retained in the final score based on pathophysiological considerations (platelet consumption in acute thrombosis) and to maintain sensitivity to the thrombotic dimension of the immunothrombosis paradigm, though future studies in populations with higher thrombocytopenia prevalence are needed to determine whether this component adds meaningful discriminative value.

### Risk stratification

**Figure 7:**
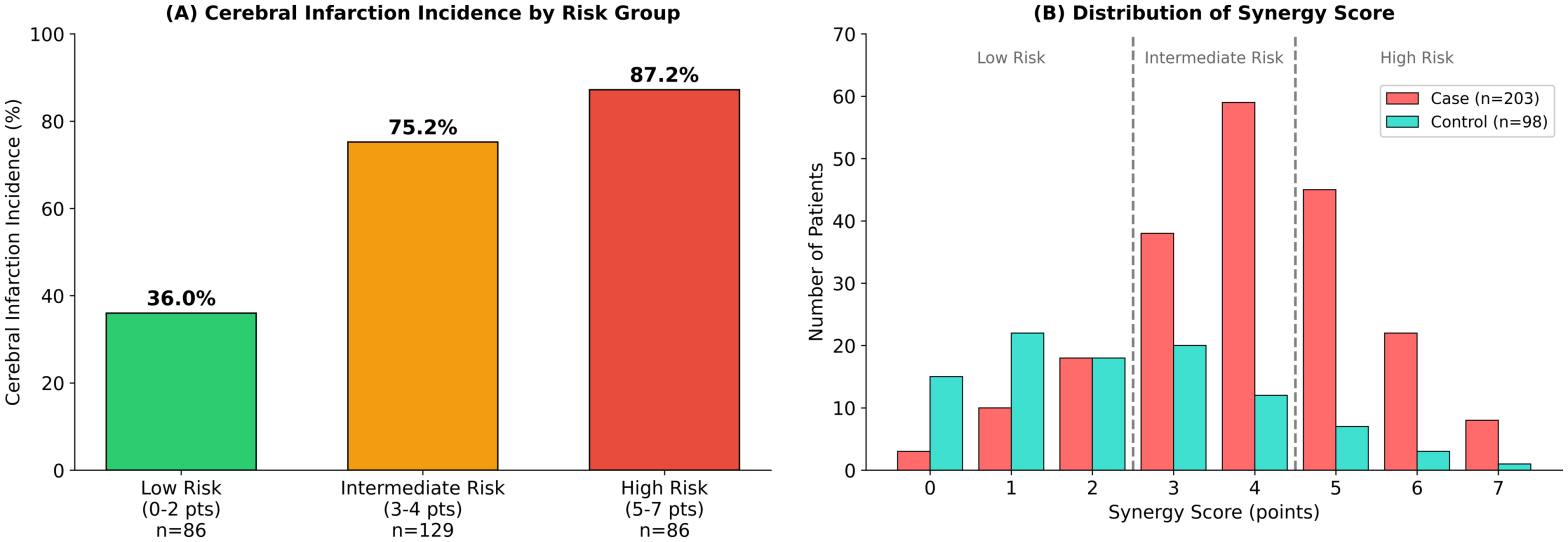
Risk stratification by inflammation-platelet synergy score. **(A)** Cerebral infarction prevalence by risk category. Low risk (0–2 points): 36.0% (31/86); intermediate risk (3–4 points): 75.2% (97/129); high risk (5–7 points): 87.2% (75/86). Cochran-Armitage test for trend: p < 0.001. Error bars represent 95% confidence intervals for proportions. **(B)** Distribution of synergy scores by case-control status. Cases (dark bars) clustered at higher scores; controls (light bars) clustered at lower scores. Numbers above bars indicate subject counts. See Table 4 for odds ratios.

Risk stratification using the inflammation-platelet synergy score revealed a pronounced gradient in cerebral infarction prevalence across categories (Table 4; Figure 7). The low-risk group (0–2 points) comprised 86 subjects, of whom 31 (36.0%) were cases and 55 (64.0%) were controls. The intermediate-risk group (3–4 points) included 129 subjects, with 97 cases (75.2%) and 32 controls (24.8%). The high-risk group (5–7 points) contained 86 subjects, comprising 75 cases (87.2%) and 11 controls (12.8%). The Cochran-Armitage test confirmed a significant linear trend in cerebral infarction prevalence across risk categories (P for trend < 0.001).

**Table 4:** Synergy score performance by risk category.

Risk category	Points	Total n	Cases n (%)	Controls n (%)	OR (95% CI)*	*p*
Low	0–2	86	31 (36.0)	55 (64.0)	Reference	—
Intermediate	3–4	129	97 (75.2)	32 (24.8)	5.38 (2.94–9.83)	<0.001
High	5–7	86	75 (87.2)	11 (12.8)	12.10 (5.40–27.15)	<0.001

P for trend (Cochran-Armitage) < 0.001. Odds ratios calculated relative to low-risk reference group using binary logistic regression. CI, confidence interval; OR, odds ratio. * = odds ratios relative to the low-risk reference group.

Compared with the low-risk reference group, the intermediate-risk group exhibited substantially elevated odds of cerebral infarction (OR = 5.38; 95% CI: 2.94–9.83; *p* < 0.001), while the high-risk group demonstrated markedly increased odds (OR = 12.10; 95% CI: 5.40–27.15; *p* < 0.001). Figure 7B illustrates the distribution of synergy scores by case-control status, demonstrating clear separation between groups with cases clustering at higher score values.

### Parameter relationships

**Figure 8:**
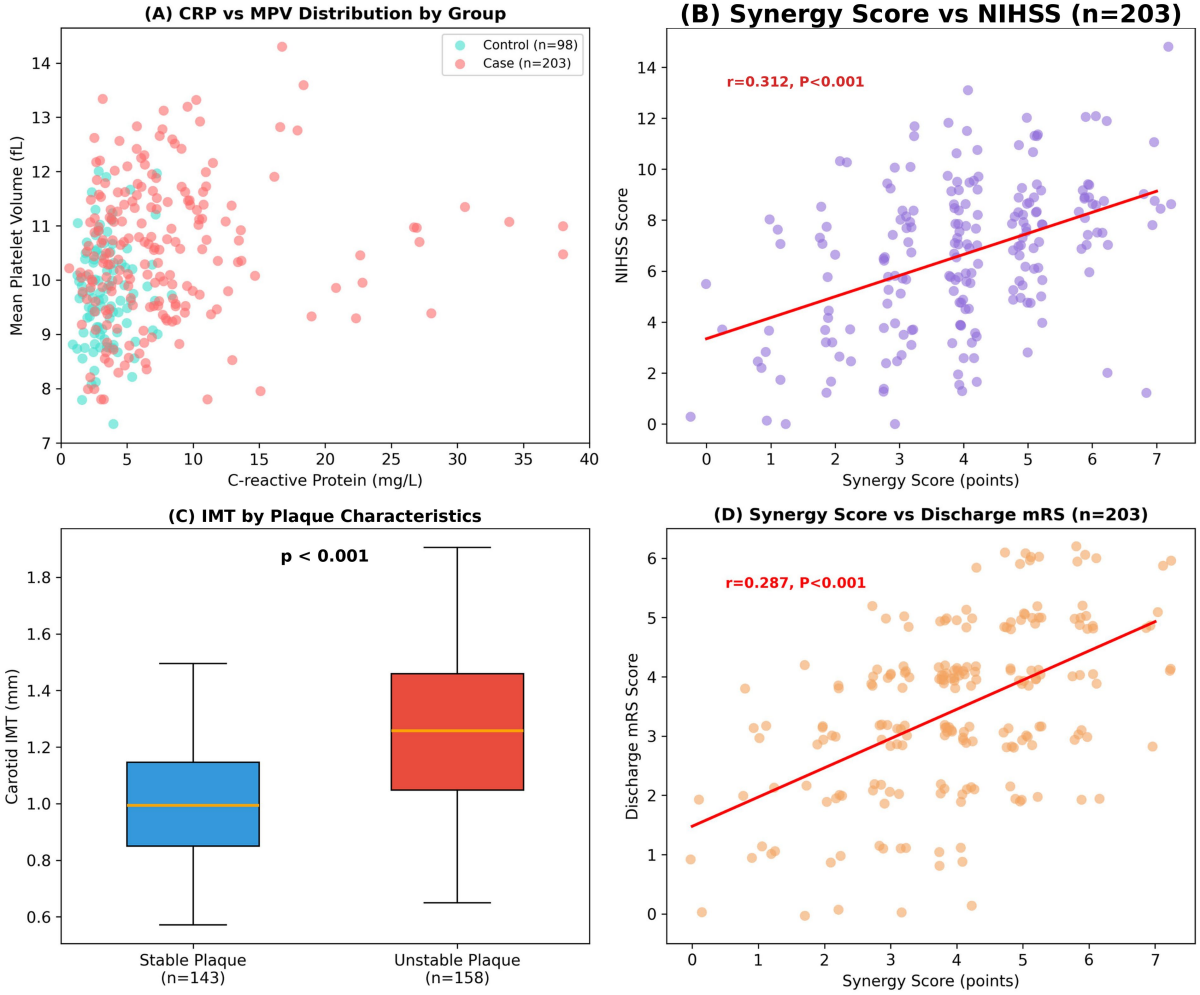
Scatter plot analysis of key parameter relationships. **(A)** Joint distribution of C-reactive protein (CRP) and mean platelet volume (MPV) by group. Cases (filled circles) clustered in the upper-right quadrant; controls (open circles) clustered in the lower-left region. Dashed lines indicate scoring thresholds (CRP > 3.0 mg/L; MPV > 10.2 fL). **(B)** Relationship between synergy score and admission stroke severity (NIHSS) among cases (*n* = 203). Spearman ρ = 0.312; *p* < 0.001. Trend line with 95% confidence band shown. **(C)** Intima-media thickness (IMT) by plaque stability classification (*n* = 301). Unstable plaques were associated with greater IMT (1.25 ± 0.28 vs. 0.98 ± 0.20 mm; *p* < 0.001; Cohen’s d = 1.11). Individual data points shown with group means ± SD. **(D)** Relationship between synergy score and discharge functional outcome (mRS) among cases (*n* = 203). Spearman ρ = 0.287; *p* < 0.001. Trend line with 95% confidence band shown. Panels **B** and **D** depict exploratory, unadjusted associations.

Scatter plot analyses characterized the relationships among key parameters and clinical outcomes (Figure 8). Panel A displays the joint distribution of CRP and MPV by group, revealing that cases clustered predominantly in the upper-right quadrant (elevated CRP and elevated MPV), whereas controls concentrated in the lower-left region. This pattern visually supports the rationale for combining these parameters in a composite score.

Panel B depicts the relationship between synergy score and admission NIHSS among cases, demonstrating that higher composite scores were associated with greater stroke severity (*ρ* = 0.312; *p* < 0.001). Panel C compares IMT values between stable and unstable plaque subtypes, showing that unstable plaques were associated with significantly greater IMT (1.25 ± 0.28 mm vs. 0.98 ± 0.20 mm; t-test: t = 8.96; df = 299; *p* < 0.001; Cohen’s d = 1.11, 95% CI: 0.84–1.38). Panel D illustrates the correlation between synergy score and discharge mRS, confirming the association between higher scores and worse functional outcomes (ρ = 0.287; *p* < 0.001).

### Clinical outcomes (exploratory secondary analysis)

The following analyses of clinical outcomes among cases are exploratory, unadjusted, and cross-sectional; they should not be interpreted as evidence of prognostic validity but rather as hypothesis-generating observations regarding the score’s potential association with stroke severity and functional outcome.

**Figure 9:**
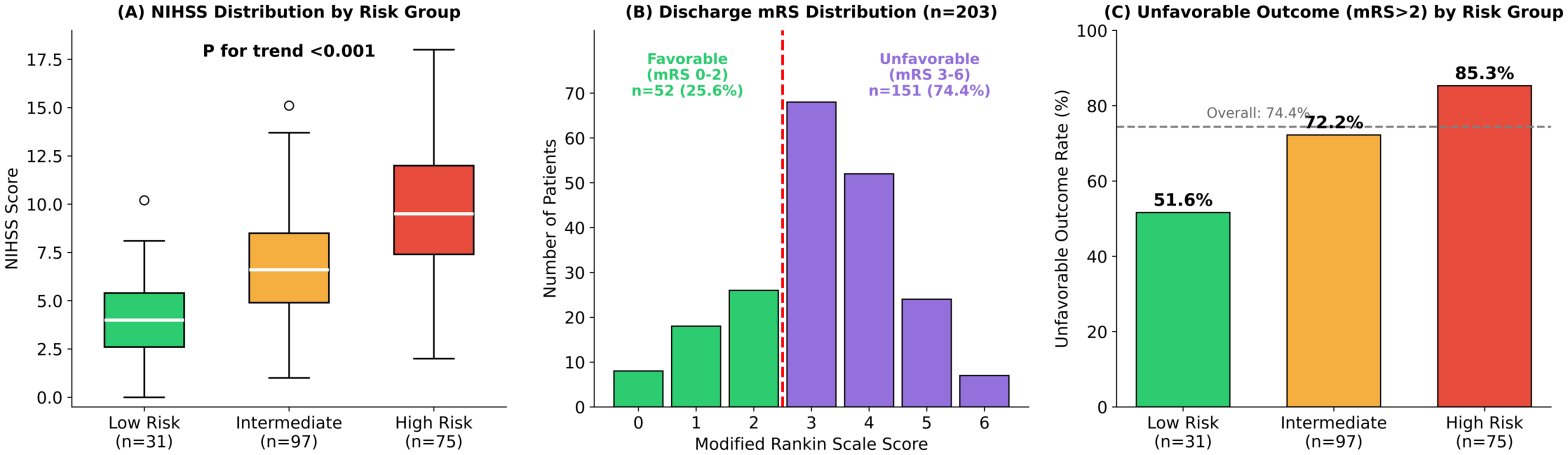
Clinical outcome analysis by risk stratification. **(A)** Admission stroke severity (NIHSS) by risk category among cases (*n* = 203). Box plots display median, interquartile range, and range. Median NIHSS: low risk = 4, intermediate risk = 7, high risk = 9. Jonckheere-Terpstra test for trend: *p* < 0.001. **(B)** Distribution of modified Rankin Scale (mRS) scores at discharge among all cases. Favorable outcome (mRS 0–2): 25.6% (52/203); unfavorable outcome (mRS 3–6): 74.4% (151/203). **(C)** Unfavorable outcome rate (mRS 3–6) by risk category. Low risk: 51.6% (16/31); intermediate risk: 72.2% (70/97); high risk: 85.3% (64/75). Cochran-Armitage test for trend: *p* < 0.001. Error bars represent 95% confidence intervals. See Table 5 for odds ratios. These are exploratory, unadjusted analyses.

Among the 203 cases with anterior circulation cerebral infarction, the inflammation-platelet synergy score demonstrated significant associations with clinical outcomes (Table 5; Figure 9). Admission stroke severity, as measured by NIHSS, increased progressively across risk strata. Median NIHSS was 4 (IQR: 2–7) in low-risk cases (*n* = 31), 7 (IQR: 4–11) in intermediate-risk cases (*n* = 97), and 9 (IQR: 6–14) in high-risk cases (*n* = 75). The Jonckheere-Terpstra test confirmed a significant trend of increasing NIHSS across ordered risk categories (P for trend < 0.001).

**Table 5:** Clinical outcomes by risk stratification among cases (*n* = 203).

Outcome	Low risk (*n* = 31)	Intermediate risk (*n* = 97)	High risk (*n* = 75)	P trend
Stroke severity (NIHSS)				
Median (IQR)	4 (2–7)	7 (4–11)	9 (6–14)	<0.001†
Mild (0–4), n (%)	18 (58.1)	32 (33.0)	14 (18.7)	
Moderate (5–15), n (%)	11 (35.5)	52 (53.6)	44 (58.7)	
Severe (≥16), n (%)	2 (6.5)	13 (13.4)	17 (22.7)	
Functional outcome (mRS)				
Favorable (0–2), n (%)	15 (48.4)	27 (27.8)	11 (14.7)	<0.001‡
Unfavorable (3–6), n (%)	16 (51.6)	70 (72.2)	64 (85.3)	
OR for unfavorable outcome (95% CI)*	Reference	2.43 (1.03–5.71)	5.53 (2.08–14.68)	
P vs. low risk	—	0.042	<0.001	

†Jonckheere-Terpstra test for ordered alternatives. ‡Cochran-Armitage test for trend. Odds ratios calculated relative to low-risk reference group. These analyses are exploratory, unadjusted, and cross-sectional. CI, confidence interval; IQR, interquartile range; mRS, modified Rankin Scale; NIHSS, National Institutes of Health Stroke Scale; OR, odds ratio. * = odds ratios relative to the low-risk reference group.

At discharge, 52 patients (25.6%) achieved favorable functional outcome (mRS 0–2), while 151 patients (74.4%) experienced unfavorable outcome (mRS 3–6). The distribution of mRS scores is displayed in Figure 9B. Unfavorable outcome rates increased monotonically across risk groups: 51.6% (16/31) in low-risk, 72.2% (70/97) in intermediate-risk, and 85.3% (64/75) in high-risk patients (Cochran-Armitage test for trend: *p* < 0.001; Figure 9C). Compared with low-risk cases, intermediate-risk cases had 2.43-fold increased odds of unfavorable outcome (OR = 2.43; 95% CI: 1.03–5.71; *p* = 0.042), and high-risk cases had 5.53-fold increased odds (OR = 5.53; 95% CI: 2.08–14.68; *p* < 0.001).

Diagnostic performance metrics for the synergy score and model calibration characteristics are summarized in Figure 5. The synergy score at the optimal cutoff (≥3 points) demonstrated balanced sensitivity and specificity for identifying cerebral infarction cases (Figure 5A). The calibration plot revealed close alignment between predicted and observed probabilities across the range of predicted risk, with points clustering along the diagonal reference line and a calibration slope of 0.92 (Figure 5B).

## Discussion

This exploratory case-control study developed and internally validated an inflammation-platelet synergy score for distinguishing patients with anterior circulation cerebral infarction from high-risk controls with carotid atherosclerosis. The combined assessment of CRP, platelet indices, and carotid characteristics demonstrated good discriminative ability (AUC = 0.824; optimism-corrected AUC = 0.812), outperforming individual biomarkers (AUC range: 0.582–0.762). A substantial gradient in cerebral infarction prevalence was observed across risk categories, from 36.0% in the low-risk group to 87.2% in the high-risk group, representing a 12-fold difference in odds. Decision curve analysis suggested positive net benefit across a range of clinically relevant threshold probabilities. These findings, while preliminary, suggest that the combined inflammation-platelet assessment captures discriminative information beyond individual markers and may warrant further evaluation in prospective settings.

The pathophysiological rationale for this approach derives from the immunothrombosis paradigm, which describes the mechanistic interplay between innate immune activation and thrombus formation in vascular disease (18, 19). Within this framework, the score components capture distinct but interconnected aspects of the immunothrombotic cascade. CRP represents the systemic inflammatory response and exerts direct prothrombotic effects through complement activation, tissue factor induction, and enhanced platelet reactivity via Fc receptor signaling (37, 39). Platelet morphological indices (MPV, PDW) reflect the thrombotic limb, with larger and more heterogeneous platelets demonstrating enhanced aggregatory and secretory capacity. Carotid parameters (IMT, unstable plaque) represent the vascular substrate upon which immunothrombotic processes operate. This conceptual distinction between acuity-based parameters (CRP, platelet indices—reflecting active inflammatory and thrombotic processes) and resource-based parameters (IMT, plaque morphology—reflecting cumulative vascular disease burden) may be relevant for clinicians interpreting the score in emergency and outpatient settings. Activated platelets release inflammatory mediators including IL-1β, CD40L, and platelet factor 4, which can amplify vascular inflammation and promote further thrombus formation (40). This bidirectional relationship may explain, in part, the improved discriminative value observed with combined assessment compared to individual markers.

The observation of elevated MPV in cases aligns with previous reports implicating enhanced platelet reactivity in stroke pathogenesis. Greisenegger et al. demonstrated in a multicenter study that elevated MPV independently predicted adverse stroke outcomes (22). Larger platelets contain greater quantities of prothrombotic mediators including thromboxane A2, serotonin, and adenosine diphosphate (41). A meta-analysis by Zheng et al. incorporating 2,390 patients confirmed that elevated MPV predicts unfavorable outcomes in acute ischemic stroke (26). The effect size for MPV in our study (Cohen’s d = 0.84) is consistent with these prior observations.

Carotid IMT and plaque characteristics exhibited strong discriminative ability, consistent with literature supporting ultrasound assessment for stroke risk evaluation (42). A meta-analysis by Lorenz et al. established that each 0.1-mm increase in common carotid IMT confers 15–18% relative risk increase for stroke (29). In our study, IMT demonstrated the highest individual AUC (0.762) and a large between-group effect size (Cohen’s d = 1.05). The association between unstable plaque and greater IMT observed here supports the link between arterial wall remodeling and plaque vulnerability (30).

The inclusion of low platelet count (<150 × 10^9^/L) in the scoring system merits specific discussion, as this component did not achieve statistical significance in multivariable analysis (*p* = 0.234). The retention was based on pathophysiological reasoning: relative thrombocytopenia in the context of acute atherothrombotic events may reflect platelet consumption at sites of active thrombus formation, a process integral to the immunothrombosis paradigm (19). However, our sensitivity analysis demonstrated that removing this component had negligible impact on discriminative performance (AUC: 0.819 vs. 0.824; DeLong *p* = 0.412). We therefore acknowledge that the current evidence does not support the independent contribution of this component, and future studies in populations with higher thrombocytopenia prevalence should re-evaluate its utility.

The moderate specificity of the synergy score at the optimal threshold (56.1%) warrants careful consideration regarding potential clinical application. This specificity level means that approximately 44% of controls would be incorrectly classified as positive, limiting the score’s value as a diagnostic tool. However, in the context of a screening-oriented application—where the clinical priority is to identify individuals at elevated risk who may benefit from closer monitoring, more aggressive risk factor management, or expedited neurovascular workup—a high-sensitivity, moderate-specificity profile may be acceptable. Higher score thresholds would improve specificity at the cost of sensitivity, and the appropriate threshold would ultimately depend on the clinical context and consequences of false-positive vs. false-negative classifications. The DCA results support that the score may offer net benefit across a range of threshold probabilities, but these estimates require prospective validation.

The work by Liu et al. demonstrating that high-sensitivity CRP modifies the prognostic value of platelet count provides support for our conceptual framework emphasizing combined assessment (31). Several practical considerations favor further evaluation: all component parameters are obtainable from routine testing (standard laboratory panels and carotid ultrasonography), the scoring system employs data-driven thresholds, and calculation is straightforward. The exploratory associations with stroke severity (NIHSS) and functional outcome (mRS) at discharge suggest the score may capture clinically meaningful variation, though these unadjusted, cross-sectional findings require confirmation in properly designed prognostic studies. The COLCOT trial, which demonstrated that low-dose colchicine reduces recurrent cardiovascular events, provides additional rationale for inflammation-targeted assessment approaches in atherothrombotic disease (43).

### Limitations

This study has several important limitations that must be considered when interpreting the findings.

First, and most importantly, the retrospective case-control design represents a fundamental methodological constraint. Case–control studies can evaluate associations and discriminative performance but cannot establish absolute risk estimates or prospective prediction. The design is subject to selection bias and spectrum bias, and the fixed case-to-control ratio does not reflect the natural prevalence of cerebral infarction in the population. The AUC and other performance metrics reported here may overestimate what would be observed in a prospective cohort setting. Accordingly, the synergy score should be considered a candidate tool requiring prospective validation rather than a clinically validated risk stratification instrument.

Second, the timing of biomarker measurement introduces a critical threat to causal interpretation. CRP and platelet indices were measured within 24 h of stroke onset for cases, and CRP elevation in particular may reflect the acute-phase response to cerebral infarction rather than a pre-existing inflammatory state. While MPV and platelet indices are generally less susceptible to acute-phase changes than CRP, the possibility of reverse causality cannot be excluded. Prospective cohort studies with pre-event baseline biomarker measurements are essential to establish whether these markers represent true antecedent risk factors. Until such evidence is available, the score should be interpreted as reflecting an association between inflammation-platelet marker profiles and cerebral infarction status, not as evidence of a causal or predictive relationship.

Third, the control group comprised high-risk individuals with carotid atherosclerosis rather than a population-based sample. While this design choice enhances clinical relevance by evaluating the score’s ability to discriminate among individuals with established vascular disease, it introduces spectrum bias and limits generalizability. The score’s performance in lower-risk populations or in individuals without established carotid atherosclerosis is unknown and may differ substantially.

Fourth, the sample size, particularly the control group (n = 98), fell short of the *a priori* estimate of 180 per group. Although the final sample provided adequate power for the primary AUC analysis, the smaller control group reduces precision of performance estimates and may limit the stability of regression coefficients. The events-per-variable ratio of 15.6 is acceptable but not generous, and overfitting remains a possibility despite the modest bootstrap optimism of 0.012.

Fifth, dichotomization of continuous biomarkers using ROC-derived thresholds, while facilitating clinical interpretation, results in information loss, reduced statistical power, and potential overfitting. Deriving thresholds within the same dataset used for model development introduces optimism bias. Future studies should consider retaining continuous variables or employing alternative approaches such as restricted cubic splines.

Sixth, the regression model did not include all potential confounders. Body mass index was not systematically recorded across all medical records and could not be included. Additional hematological markers (e.g., neutrophil-to-lymphocyte ratio, fibrinogen) were beyond the predefined scope of this investigation. While smoking status and statin use were included and showed no significant independent associations, residual confounding from unmeasured variables cannot be excluded.

Seventh, the contribution of low platelet count (<150 × 10^9^/L) was limited, as this threshold was exceeded by most participants (91.4%), and this component did not achieve statistical significance in multivariable analysis. The sensitivity analysis confirmed minimal impact of this component on overall score performance.

Eighth, the comparison of the composite score’s AUC against individual component AUCs is inherently biased in favor of the composite. While this is a standard analytic approach for evaluating whether combination improves discrimination, it does not constitute rigorous assessment of incremental value. Net reclassification improvement or integrated discrimination improvement analyses would provide complementary evidence but were not performed in this study.

Ninth, the associations between the synergy score and clinical outcomes (NIHSS, mRS) were exploratory, unadjusted, and cross-sectional. These analyses do not constitute prognostic validation and should not be interpreted as evidence that the score predicts stroke severity or functional outcome.

Tenth, the study was conducted at a single center, and external validation in geographically, ethnically, and clinically diverse populations is necessary before any generalization of the findings.

Future investigations should prioritize prospective cohort validation with pre-event biomarker assessment, external validation across multiple centers, and evaluation of whether score-guided interventions improve clinical outcomes. Additionally, incorporation of novel biomarkers such as high-sensitivity cardiac troponin or lipoprotein-associated phospholipase A2 may further enhance discriminative performance.

## Conclusion

In this exploratory case-control study, the inflammation-platelet synergy score demonstrated good discriminative ability for anterior circulation cerebral infarction, outperforming individual biomarkers. The composite score was associated with a pronounced gradient in cerebral infarction prevalence across risk categories and showed exploratory associations with stroke severity and functional outcome. However, the retrospective case-control design, post-event biomarker measurement, and absence of external validation preclude clinical application at this stage. These findings should be considered hypothesis-generating and support the concept that combined assessment of inflammatory and platelet markers within the immunothrombosis framework may capture discriminative information beyond individual markers. Prospective cohort studies with pre-event biomarker measurement and external validation are required to determine whether this approach has genuine predictive and clinical utility.

## Data Availability

The original contributions presented in the study are included in the article/Supplementary material, further inquiries can be directed to the corresponding author.
