# Supplementary material for: Development and internal validation of an inflammation-platelet synergy score for anterior circulation cerebral infarction: an exploratory case-control study
Source: Front Neurol. 2026 Jul 16;17:1805305. doi: 10.3389/fneur.2026.1805305 (PMC13421415; doi:10.3389/fneur.2026.1805305)
Supplement: Supplementary file 1 [file Table_1.DOCX]

**Supplementary Table S1. Sensitivity Analysis: Score Performance With and Without Platelet Count Component.**

| **Score Version** | **Components** | **Range** | **AUC (95% CI)** | **Optimism-corrected AUC** | **DeLong P** |
| --- | --- | --- | --- | --- | --- |
| Full score (6 parameters) | CRP, MPV, PDW, PLT, Unstable plaque, IMT | 0–7 | 0.824 (0.781–0.867) | 0.812 | Reference |
| Modified score (5 parameters) | CRP, MPV, PDW, Unstable plaque, IMT | 0–6 | 0.819 (0.774–0.864) | 0.808 | 0.412 |

*AUC = area under the receiver operating characteristic curve; CI = confidence interval; CRP = C-reactive protein; IMT = intima-media thickness; MPV = mean platelet volume; PDW = platelet distribution width; PLT = platelet count.*
